# A Review of Advancements and Challenges in Liver Segmentation

**DOI:** 10.3390/jimaging10080202

**Published:** 2024-08-21

**Authors:** Di Wei, Yundan Jiang, Xuhui Zhou, Di Wu, Xiaorong Feng

**Affiliations:** Department of Radiology, The Eighth Affiliated Hospital of The Sun Yat-sen University, No. 3025, Middle Shennan Road, Shenzhen 518033, China; weid6@mail.sysu.edu.cn (D.W.); jiangyd7@mail.sysu.edu.cn (Y.J.); zhouxuh@mail.sysu.edu.cn (X.Z.)

**Keywords:** liver segmentation, medical imaging, deep learning, convolutional neural networks, fully, convolutional networks, U-Net, automated segmentation

## Abstract

Liver segmentation technologies play vital roles in clinical diagnosis, disease monitoring, and surgical planning due to the complex anatomical structure and physiological functions of the liver. This paper provides a comprehensive review of the developments, challenges, and future directions in liver segmentation technology. We systematically analyzed high-quality research published between 2014 and 2024, focusing on liver segmentation methods, public datasets, and evaluation metrics. This review highlights the transition from manual to semi-automatic and fully automatic segmentation methods, describes the capabilities and limitations of available technologies, and provides future outlooks.

## 1. Introduction

The liver is a crucial organ in the human body with a complex anatomical structure and valuable physiological functions [1,2]. With the rapid development of medical imaging technologies, liver scans generate large amounts of images. Liver segmentation technologies have become increasingly vital, aiding clinicians in clinical diagnosis, disease monitoring, and surgical planning. As one of the largest internal organs performing complex functions, the liver can present a variety of pathological changes exhibiting high degrees of heterogeneity on medical images, which makes liver segmentation challenging [3,4,5].

Accurate segmentation is important for the diagnosis and severity assessment of liver diseases (e.g., cancer and cirrhosis). In addition, by precisely locating and quantifying pathological liver sections, doctors can develop more personalized and effective treatment plans. Before liver surgeries such as resection and transplantation, precise segmentation helps doctors understand the patient-specific morphological structure of the liver, including the location and size of blood vessels and tumors. This enables precise surgical planning, reduces surgical risks, and increases operation success rates [6,7,8]. During radiation therapy, accurate liver segmentation helps clinicians deliver radiation doses precisely and sufficiently to tumor cells while minimizing damage to surrounding healthy tissues [8]. For patients undergoing treatment for liver diseases, regular liver scanning, segmentation, and analysis aid in the monitoring of disease progression, assessment of treatment effectiveness, and timely adjustment of treatment strategies. For liver transplant patients, liver segmentation technology can be used to process large datasets and optimize decision-making in donor matching, potentially reducing mortality by reducing waitlist times. Additionally, it can aid the prediction of transplant recipient and graft survival rates and the identification of risk factors for disease recurrence and complications [9]. In medical research and education, liver segmentation technology can be used to demonstrate the complexity of the liver and its physiological functions, promoting the development of new treatment methods and technologies [3,10].

In recent years, liver segmentation has progressed remarkably from manual to semi-automatic and fully automatic methods, with deep learning, particularly convolutional neural networks (CNNs), playing a pivotal role. These advancements have significantly improved the efficiency and accuracy of liver segmentation, enabling more reliable clinical applications [3,5]. Several challenges, however, remain. Individual variations in liver shape and size make the development of universally applicable algorithms difficult. Additionally, public datasets and standardized evaluation metrics are lacking [5]. Addressing these issues in the continued development of liver segmentation technologies will enhance their accuracy and utility in clinical practice, research, and education, leading to better patient outcomes and advances in medical imaging. This article provides a retrospective review of liver segmentation technology, including its development, available public databases, and benchmarking standards. It also offers insights into future technological trends.

## 2. Materials and Methods

The authors systematically searched the PubMed, IEEE Xplore, Google Scholar, and ScienceDirect databases for liver segmentation studies published between 2014 and 2024. To ensure comprehensive coverage of the topic, the keywords “liver segmentation”, “medical imaging”, “deep learning”, “liver segmentation dataset”, “liver segmentation metrics”, and “convolutional neural networks” were used. Initially, 97 articles were identified. Conference abstracts, non-peer-reviewed articles, and articles lacking the required depth and breadth of content (i.e., sufficiently detailed and comprehensive reporting of literature-based background, analysis, and/or results) were excluded, leaving 43 high-quality articles that met the criteria for detailed analysis. We summarized these articles, recorded the public databases used in the studies and their advantages and disadvantages, documented the evaluation metrics used and their strengths and weaknesses, and identified key technological milestones in liver segmentation from the articles’ background sections.

## 3. Results

### 3.1. Public Datasets

**Figure 1:**
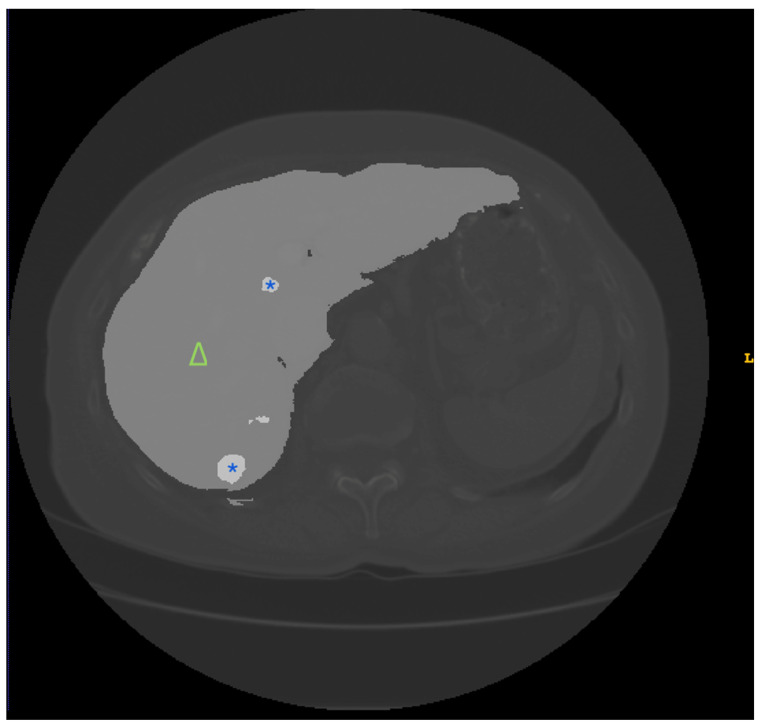
Example images from the LiTS dataset [11]. LiTS, Liver Tumor Segmentation Challenge. The triangle (∆) represents the liver region, and the asterisk (*) represents the lesion area.

**Figure 2:**
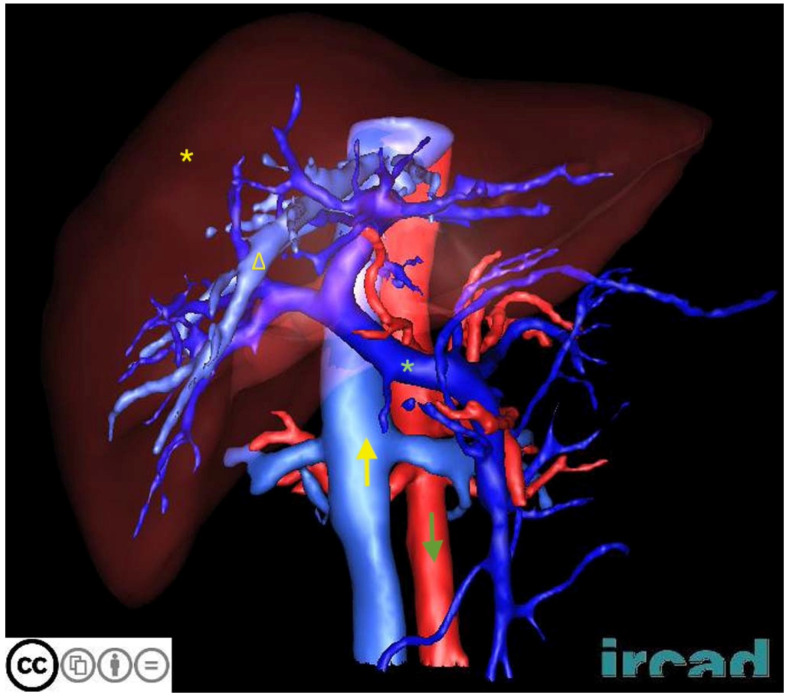
Example image from the 3DIRCADb dataset [12]. 3DIRCADb, 3D Image Reconstruction for Comparison of Algorithm Database. The yellow asterisk (*) represents the liver, the yellow hollow triangle (∆) represents the hepatic vein, the yellow upward arrow (↑) represents the inferior vena cava, the green asterisk (*) represents the portal vein, and the green downward arrow (↓) represents the abdominal aorta.

Several important public datasets for liver segmentation research and development are available (Table 1). The most notable datasets are the Liver Tumor Segmentation Challenge (LiTS; Figure 1) and the 3D Image Reconstruction for Comparison of Algorithm Database (3DIRCADb; Figure 2). The LiTS dataset contains 201 abdominal computed tomography (CT) volumes with annotations for liver and liver tumor segmentation. It was established to facilitate the development of automatic segmentation technologies and to address complexities such as changes in the lesion-to-background contrast, the diversity of lesion types, and changes in liver tissue signals caused by chronic liver diseases. However, the variety of liver lesions included complicates algorithm development. The 3DIRCADb contains CT scan data, including that on liver and liver tumor segmentation, from 20 patients. It is used widely in medical image processing, especially for the automatic recognition and analysis of liver structures and lesions. However, a limited number of case types are represented in the small sample.

**Figure 3:**
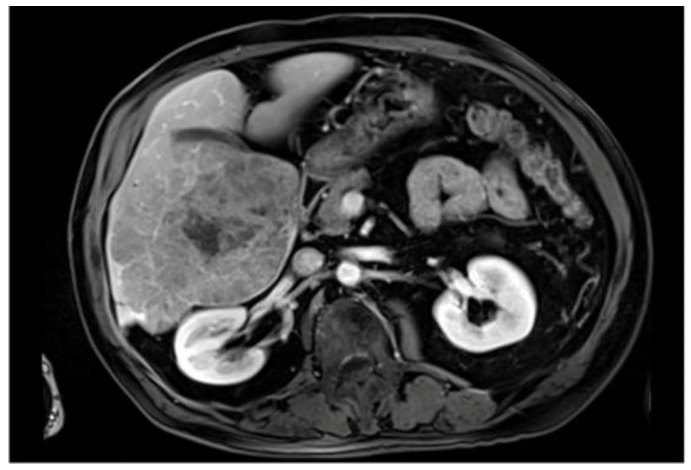
Example image from the ATLAS dataset [21]. ATLAS, Tumor and Liver Automatic Segmentation.

**Figure 4:**
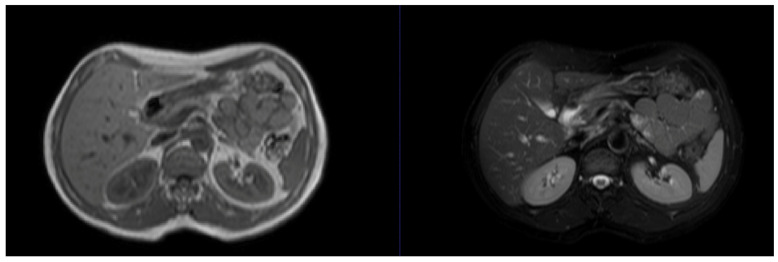
Example images from the CHAOS dataset [22]. CHAOS, Combined (CT-MR) Healthy Abdominal Organ Segmentation.

The classic Segmentation of the Liver (SLIVER07) dataset contains CT images of diseased livers and, like the 3DIRCADb, is used widely to evaluate and train liver segmentation algorithms [4,5,8,13,14,15,16,17]. However, it is an older dataset that may not include newly identified lesion types or images acquired using the most current technology. The recently published Tumor and Liver Automatic Segmentation (ATLAS) dataset (Figure 3) [18] focuses on liver and tumor segmentation by contrast-enhanced magnetic resonance imaging (MRI), especially for inoperable hepatocellular carcinoma. It is the first public dataset to offer annotations for this context, with the aim of optimizing contouring in liver cancer treatment planning. However, the compatibility of this newer dataset with broader applications remains to be validated. The Combined (CT-MR) Healthy Abdominal Organ Segmentation (CHAOS) dataset (Figure 4) contains healthy kidney, liver, and spleen imaging data from 80 patients in Digital Imaging and Communications in Medicine format, with ground-truth masks annotated by certified radiologists to ensure accuracy and reliability [8,19,20]. This dataset supports research on cross-modality medical image processing, but the small patient sample size may limit the generalizability of models and large-scale evaluation, and the provision of data from only healthy organs may restrict its applicability in disease-specific research.

These public datasets have been crucial in advancing liver segmentation technology, but each has its limitations. More comprehensive and representative datasets are needed to meet evolving needs in this field and improve the diagnosis and treatment of liver diseases.

### 3.2. Evaluation Standards

Several metrics are commonly used to evaluate and compare liver segmentation algorithm performance (Table 2). They consist primarily of similarity-based, distance-based, and pixel-wise accuracy metrics [13,14,15,17].

The dice similarity coefficient (DSC; Equation (1)) is commonly used to assess segmentation quality, as it reflects the similarity between predicted and ground-truth segmentations. Values closer to 1 reflect more precise segmentation. The DSC is an intuitive measure that is well-suited for the quantification of model performance improvements. When the sizes of the two segmented areas are extremely inequal, the DSC may be misleading because it imposes a greater penalty for errors in smaller areas.

(1) $$\mathrm{DSC}=\frac{2\times \left|A\cap B\right|}{\left|A\right|+\left|B\right|}\;$$

where *A* represents the predicted segmentation region and *B* represents the ground-truth segmentation region.

The Jaccard index (JI; Equation (2)), also known as the intersection over union coefficient, is the ratio of the intersection to the union of the predicted and true segmentations. Similar to the DSC, the JI measures the overlap between two sets and is commonly used to gauge the similarity of samples. It is sensitive to noise and minor deviations in segmentation boundaries, which may cause fluctuations in its value.

(2) $$\mathrm{Jaccard}\;\mathrm{index}=\frac{\left|A\cap B\right|}{\left|A\cup B\right|},\;$$

where *A* represents the predicted segmentation region and *B* represents the ground-truth segmentation region.

The accuracy (Equation (3)), sensitivity (Equation (4)), and specificity (Equation (5)) metrics are used to assess a segmentation algorithm’s ability to detect liver tissue and lesions. The accuracy reflects the proportion of correctly classified voxels out of the total number of voxels. The sensitivity and specificity reflect the algorithm’s ability to identify true-positive (i.e., liver regions) and true-negative (i.e., non-liver regions) samples, respectively. These metrics evaluate algorithm performance from different perspectives: the accuracy is a measure of the overall correctness, sensitivity focuses on the identification of areas of interest, and specificity focuses on the exclusion of non-target areas. Used alone, they may not be sufficient for comprehensive performance evaluation, as high sensitivity can be accompanied by low specificity and vice versa.

(3) $$\mathrm{Accuracy}=\frac{\mathrm{TP}+\mathrm{TN}}{\mathrm{TP}+\mathrm{TN}+\mathrm{FP}+\mathrm{FN}}\;$$

(4) $$\mathrm{Sensitivity}=\frac{\mathrm{TP}}{\mathrm{TP}+\mathrm{FN}},\;$$

(5) $$\mathrm{Specificity}=\frac{\mathrm{TN}}{\mathrm{TN}+\mathrm{FP}},\;$$

where *TP* represents the number of true-positive samples, *TN* represents the number of true-negative samples, *FP* represents the number of false-positive samples, and *FN* represents the number of false-negative samples.

The volume overlap error (VOE; Equation (6)) is an expression of the proportion of the non-overlapping region to the total combined region of the predicted and ground-truth segmentations. It is thus a reflection of the overall segmentation quality, with lower values reflecting greater accuracy. The VOE is suitable for the assessment of segmentation precision for large-volume structures but may not be accurate for small volumes and can be affected by incidental errors.

(6) $$\mathrm{VOE}=1-\frac{\left|A\cap B\right|}{\left|A\cup B\right|},\;$$

where *A* represents the predicted segmentation region and *B* represents the ground-truth segmentation region.

The relationship between the VOE and the JI is straightforward; the former is essentially the complement of the latter (Equation (7)):

(7) VOE=1−Jaccard index.

The relative volume difference (RVD; Equation (8)) quantifies the error between the predicted and actual volumes and is often used with the VOE. The complexities of detailed shapes may be overlooked with RVD calculation, and noise (e.g., that from small isolated regions) can significantly impact volume calculations and thus the accuracy of the RVD.

(8) $$\mathrm{RVD}=\frac{\left|A\right|-\left|B\right|}{\left|B\right|},\;$$

where *A* represents the predicted segmentation region and *B* represents the ground-truth segmentation region.

Boundary distance-based metrics, such as the average symmetric surface distance (ASSD; Equation (9)) and maximum symmetric surface distance (MSSD; Equation (10)), are also used to quantify discrepancies between predicted and true segmentation and, in particular, the accuracy of segmentation boundaries.

(9) $$\mathrm{ASSD}=\frac{1}{\left|S_{A}\right|+\left|S_{B}\right|}\left(\sum_{x\in S_{A}}d\left(x,S_{B}\right)+\sum_{y\in S_{B}}d\left(y,S_{A}\right)\right),\;$$

(10) $$\mathrm{MSSD}=\max\left(\max_{x\in S_{A}}d\left(x,S_{B}\right),\max_{y\in S_{B}}d\left(y,S_{A}\right)\right),\;$$

where *S_A_* and *S_B_* are the surfaces of the predicted and ground-truth segmentations, respectively; *d*(*x*, *S*) represents the shortest distance from point *x* to surface *S*; *x* is a point on the predicted segmentation surface; and *y* is a point on the ground-truth segmentation surface.

The combined use of evaluation metrics provides a comprehensive understanding of segmentation algorithm performance, and the choice of which metrics to use depends on the specific application needs and experimental design. In research and clinical applications, the use of multiple metrics is often required to comprehensively evaluate the effectiveness and suitability of the algorithms.

## 4. Development and Evolution of Liver Segmentation Technology

### Early Methods

Initially, liver segmentation relied on radiologists’ or technicians’ manual delineation of liver contours on CT or MRI images. Although considered the gold standard in clinical practice and research, this approach was time-consuming, depended heavily on operators’ experience and expertise, and was not suitable for busy clinical environments. The development of semi-automatic and fully automatic segmentation methods has significantly improved liver segmentation. These methods can greatly reduce workload but may require manual intervention in the presence of complex or atypical anatomical variations [3,8,15,16,20].

## 5. Threshold and Region-Growing Methods

Threshold segmentation is performed by setting a specific grayscale threshold distinguishing liver from non-liver tissues. It is simple to execute but sensitive to the threshold setting, is heavily dependent on image quality, and is susceptible to noise and contrast variations. The region-growing algorithm, based on pixel similarity, starts from a seed point and gradually expands to similar surrounding pixels [23]. This method adapts better to local changes in image characteristics than does threshold segmentation, enhancing the accuracy and suitability for complexly shaped structures.

## 6. Edge- and Shape-Based Methods

Edge-based segmentation can complement region growing by providing precise organ boundary definitions when edges are clear, capturing complex structures. More recently developed shape-based segmentation methods, such as statistical shape and active-contour modeling, use prior shape knowledge to guide image segmentation, improving the recognition of complex structures [5,24].

The development of statistical and probabilistic graphical models has greatly enhanced liver image analysis capabilities. With greater robustness and better generalization, these models more effectively handle complex structures, especially those on medical images with complex backgrounds and noise, enabling more accurate and reliable segmentation than that achieved with traditional edge- and region growing-based methods. They have thus improved the accuracy of liver disease diagnosis and treatment plan development [5,8]. Each of these techniques has its own characteristics and limitations. For example, statistical shape models use statistical information to process local image regions, providing a certain degree of robustness to noise, but they are computationally complex and require substantial computing resources. Active contour models involve complex parameter tuning and are sensitive to initial contour positions, which may lead to errors. Over time, these methods have merged and evolved to meet the more complex demands of liver segmentation. In practical applications, they are often combined to leverage their respective advantages, improving segmentation accuracy and efficiency.

## 7. Deep Learning and Fully Convolutional Networks

The development of fully convolutional networks (FCNs) was a major step in the evolution of liver segmentation technology. FCNs accept arbitrarily sized image inputs and make pixel-level predictions based on learned features. They also provide end-to-end segmentation, making them effective for liver applications. Long et al. [25] demonstrated the application of FCNs in semantic segmentation, laying the foundation for their use with medical images. However, these networks may perform poorly for images with small objects or with numerous details.

Residual networks (ResNets) enable the training of deeper models without increasing the training difficulty, thereby improving model performance [26]. Litjens et al. [27] described the leveraging of their deep architecture and ability to learn detailed features to improve the accuracy and robustness of liver segmentation. ResNets better delineate liver boundaries and detect liver lesions, which is critical for accurate diagnosis and treatment planning. However, the complexity of their structures can lead to overfitting (i.e., good performance with training data but poor generalization) with small datasets. Additionally, due to their deep network structures, ResNets require more computational resources [e.g., graphics processing unit (GPU) and central processing unit capacities and memory] for training and inference. Finally, their numerous components, including residual connections and bottleneck architecture, make tuning complex and necessitate extensive experimentation to find optimal parameter combinations.

## 8. Advances with U-Net and Variants

U-Net is a CNN that effectively captures contextual information and fine details from images through symmetrical encoding and decoding paths [28,29,30,31]. Wang et al. [29] developed a generalized U-Net network for automated liver segmentation and biometry from CT and MRI images that showed enhanced robustness when trained on a dataset with variations in liver size, shape, and pathology. This approach enabled accurate and reliable liver segmentation, facilitating clinical assessment and treatment planning [29]. However, U-Net–based segmentation is challenging when small and irregularly shaped tumors have intensities similar to those of surrounding tissues on CT images [32]. In addition, in terms of segmentation efficiency and accuracy, U-Net may not perform as well as more complex models for very large images, which require more computational resources, increased processing times, and specialized algorithms, or for more complex background differentiation.

The densely connected convolutional network (DenseNet) has been applied to liver segmentation with notable success. It connects each layer to every other layer in a feed-forward fashion, enhancing feature reuse and reducing the number of parameters. This architecture mitigates the vanishing gradient problem and improves information flow across the network, leading to better feature propagation and stronger gradient flow [33,34,35]. DenseNet is particularly effective for liver segmentation because it can handle complex and diverse liver structures with high efficiency. Its ability to produce high-resolution segmentation maps makes it a valuable tool in medical image analysis. However, its computational cost and memory consumption are large and the training times are long, especially when handling large amounts of three-dimensional (3D) data.

SegNet has an encoder-decoder structure that is relatively simple and easy to implement, making it suitable for pixel-level image segmentation. The encoder uses max-pooling layers to downsample input images and capture features at multiple scales. The decoder then uses indices from the max-pooling layers for upsampling, avoiding fully connected layers. This approach reduces the number of parameters while retaining spatial information, which is beneficial for fine segmentation tasks. As it does not need to store full-size feature maps, SegNet consumes less memory than do other fully convolutional networks (e.g., FCNs), making it suitable for use in resource-constrained environments. SegNet provides relatively accurate liver segmentation results and handles edge details well [28,36]. Its accuracy, however, may not match that of models with more complex network structures (e.g., U-Net and DenseNet) for some intricate medical image segmentation tasks. In addition, SegNet training requires more time, especially on large-scale datasets, due to the network’s depth, and large amounts of data are needed to avoid overfitting. SegNet effectively segments larger objects, but its performance for smaller objects or areas with complex details may not be as good as that of more detailed models [33,34,37].

V-Net is a U-Net variant designed specifically for 3D image (e.g., CT and MRI) data. It directly processes such data and uses skip connections to fuse features from the encoder and decoder, retaining more spatial information and improving segmentation boundary accuracy [38,39,40]. Due to its deep structure and residual connections, V-Net can extract high-level features from images, aiding in the precise segmentation of complex organ structures. It also performs multiscale feature extraction. The use of 3D convolutions, however, requires substantial computational resources (e.g., GPU capacity and memory) for training and inference. In addition, V-Net’s complexity and large number of parameters result in long training times. On small-scale datasets, this complexity can lead to overfitting, necessitating data augmentation and regularization. V-Net contains numerous hyperparameters (e.g., kernel size, number of layers, and learning rate), making tuning complex. Finally, its 3D convolutions and intricate network structure make implementation and debugging more challenging than for two-dimensional networks.

Three-dimensional U-Net is more capable than U-Net of handling 3D medical imaging data. Its use of 3D contextual information improves segmentation accuracy [39]. Like other complex network structures with numerous parameters, 3D U-Net has high computational resource requirements, long training times, and a tendency to overfit on small datasets.

R2U-Net combines the strengths of U-Net, residual connections, and recurrent neural networks. It alleviates the vanishing gradient problem in deep networks via residual connections while enhancing feature extraction capabilities via recurrent mechanisms [41]. Residual connections increase the effectiveness of information flow between layers, improving learning and enabling feature reuse. These features are particularly advantageous for the precise segmentation of complex organs such as the liver. Recurrent convolution enhances feature representation through multiple convolution operations, capturing richer contextual information and thereby improving segmentation accuracy [40,41]. With the incorporation of the skip connections from U-Net, R2U-Net retains high-resolution feature information and thus performs exceptionally well in detailed and complex liver segmentation tasks. R2U-Net can adapt to variations in liver sizes and shapes and lesion area types and has demonstrated strong robustness and generalization capabilities.

## 9. Integration of Emerging Technologies

The application of neural network technology has brought revolutionary advancements to liver segmentation. These networks can automatically learn complex image features from large amounts of training data, achieving high-precision liver segmentation. Three-dimensional segmentation technologies in particular provide highly accurate and comprehensive information about liver volume and morphology [26,42].

Multimodal deep learning, in which data from different imaging modalities are combined to leverage modality strengths (e.g., the high resolution of CT and high contrast of MRI), provides more comprehensive diagnostic information than do methods based on data from single modalities, improving the accuracy and reliability of disease diagnosis [18,39,40]. These approaches also improve lesion identification and localization, especially in complex clinical cases, and predictive performance. Multimodal artificial intelligence (AI) technology effectively integrates information from different sources, providing more accurate support for diagnosis and treatment [20,43,44]. However, the precise temporal and spatial alignment of multimodal data can be technically challenging, particularly when dynamic imaging data or data from different devices are involved. In addition, multimodal data processing and analysis typically require more computational resources and time, which may limit the real-time application of these approaches in clinical practice.

## 10. Key Technological Milestones

Cascaded models typically consist of multiple network stages or levels, with each stage involving the extraction of more detailed information from the previous stage’s output. In liver segmentation, the approximate location and shape of the liver are identified in the first stage, and boundaries and details are refined in subsequent stages to improve segmentation accuracy [17,19,45]. This type of network is particularly suitable for liver segmentation on MRI images. Although cascaded models may appear to be computationally complex, they enhance efficiency by avoiding repetitive fine-grained analysis over the entire image and focusing on areas requiring detailed processing. Due to their multiple processing stages, cascaded models may require more computational resources and be challenging to use in resource-constrained environments. In addition, the training of cascaded models is more complex than the training of single networks, requiring careful design of the network architecture and training strategy for each stage. Finally, stage performance may be interdependent, and errors in earlier stages could be amplified in subsequent stages.

The hierarchical processing approach of cascaded models and comprehensive information processing capabilities of multimodal deep learning methods can theoretically complement each other, especially for the analysis of medical images with different characteristics and those depicting complex anatomical structures. This combination could be explored further in future research.

The development of liver segmentation has been based on technological advancements and reflects the interplay among computational techniques, algorithm design, and medical needs. Each step of progress has built upon the previous one, and such progress has often resulted from collaboration among researchers in multiple fields. An overview of liver segmentation techniques is provided in Table 3.

## 11. Discussion

Liver segmentation is a crucial component of medical image processing for liver diseases, especially in radiological diagnostics and surgical planning. Its accuracy is affected by factors such as individual variations in liver location and morphology, reduced image quality due to suboptimal equipment or operator skill, liver texture and morphology changes caused by diseases such as cirrhosis and tumors, and lack of sufficient contrast between tumor and normal liver tissue. Increasingly advanced segmentation techniques have been developed with the aim of overcoming such issues, but limitations and challenges persist. In complex clinical scenarios, precise fully automatic segmentation often requires lengthy computation times and significant computational power, posing challenges for real-time surgical planning.

The application of deep learning has brought unprecedented progress in liver segmentation, but the performance of deep learning models depends heavily on the use of large amounts of high-quality training data. Public datasets have been instrumental in advancing liver segmentation technology by providing the diverse and high-quality data needed for deep learning model training and evaluation, but each dataset has its limitations. Future research should focus on the development of more comprehensive and representative datasets to meet evolving needs for liver segmentation. Additionally, the cost of acquiring high-quality annotated data, especially in the medical field, is high, and the complexity of medical imaging data and limited availability of datasets can lead to deep learning models being prone to overfitting.

Evaluation standards for liver segmentation are crucial for the development and optimization of segmentation algorithms because they directly impact how algorithm performance is measured and compared. Currently used metrics can reflect the statistical performance of algorithms but may not accurately represent clinical relevance, such as whether segmentation results help to improve surgical planning or treatment outcomes. Due to the lack of a unified evaluation protocol and standard datasets, the comparison of findings from different studies may be biased. Thus, the creation of more widely accepted, comprehensive (in terms of disease type and case complexity) public datasets and the development of benchmark testing are essential to ensure sound comparison of the performance of different algorithms under the same conditions.

Future research on liver segmentation technologies should also focus on the enhancement of algorithm robustness and generalization, multimodal data fusion, real-time segmentation and adaptation to low-resource environments, and the integration of emerging technologies. To address model generalization issues across datasets, techniques such as data augmentation, transfer learning, and model regularization can be employed. Collaborative efforts to build large-scale, multi-center datasets will also enhance model generalization. Multimodal data fusion can be achieved by developing multimodal neural networks and joint feature-learning frameworks. For real-time and low-resource applications, the design of lightweight models and application of model compression and hardware optimization techniques are crucial. The incorporation of emerging technologies such as quantum computing, blockchain, and federated learning holds promise for further improving the accuracy and scope of liver segmentation technology. These research directions and innovations will lead to the generation of more efficient, accurate, and widely applicable liver segmentation solutions.

The rapid development of AI technology, including the widespread application of deep learning and machine learning, has significantly increased the demand for computational resources and has driven the rapid enhancement of hardware capabilities. With the advancement of cutting-edge technologies such as quantum computing and neural network chips, we may be entering a new era of computing. This will enable end devices to handle more complex AI tasks in real time, meeting the requirements for high-speed, large-volume data processing at medical terminals. Such advances will be able to accommodate more complex liver segmentation algorithms. In this context, the protection of patient privacy becomes especially important, and data encryption is a crucial technical measure. This process involves two key aspects. First, robust encryption algorithms must be applied to protect all patient data during storage and transmission, ensuring that unauthorized individuals cannot access the data in the event of a breach. Second, authentication mechanisms should be applied to prevent unauthorized access to patient data. Moreover, data computation and storage should occur locally, at the institutions where patients undergo examination, without uploading to the cloud. Technology providers, and any other companies providing technological support, should offer algorithmic support only to the institutions and must be strictly prohibited from accessing patient data without authorization. Furthermore, it is essential to establish and adhere to strict privacy policies and legal regulations, along with effective oversight mechanisms, to ensure that all actions are compliant and lawful, ensuring patient privacy and enhancing the efficiency of medical work.

AI will continue to be essential to clinicians in their practice. AI can provide preliminary analyses and identify areas of concern, while doctors can offer expert interpretations, correct any inaccuracies, and make final diagnoses. This partnership leverages the strengths of machine and human intelligence, enhancing the overall efficiency of clinical work and ultimately benefiting humanity.

## 12. Conclusions

The development and application of liver segmentation technologies are of great importance to enhance the diagnosis, treatment, and management of liver diseases and thus constitute a key research direction in the field of medical imaging. With advancements in AI and machine learning technologies, liver segmentation is becoming more precise and automated, enabling the provision of better medical services to patients.

## Figures and Tables

**Table 1:** Major Public Datasets for Liver Segmentation.

Dataset	Content	Main Advantages	Main Disadvantages
LiTS	201 abdominal CT scans with annotations for liver and liver tumor segmentation	Rich data, especially suitable for complex cases	Diversity of liver lesions represented may complicate algorithm development
3DIRCADb	CT scans from 20 patients with annotations for liver and liver tumor segmentation	Detailed 3D reconstruction data aid the development of segmentation algorithms for complex liver structures	Small sample with limited case types
SLIVER07	CT images of diseased livers	Useful for algorithm evaluation and comparison	Older datasets that may lack recently recognized lesion types and technologically up-to-date images
ATLAS	Annotated CE-MRI data, particularly for inoperable HCC	First dataset of its kind, suitable for the optimization of contouring in liver cancer treatment planning	Newer datasets requiring validation of compatibility for widespread use
CHAOS	Abdominal (kidney, liver, and spleen) CT and MRI scans from 80 patients in DICOM format with ground-truth masks annotated by certified radiologists	Promotes multi-modality imaging research and provides data on healthy organs that are useful for benchmarking	Small sample and lack of pathological information, may be insufficient for model training for pathology detection

LiTS, Liver Tumor Segmentation Challenge; CT, computed tomography; 3DIRCADb, 3D Image Reconstruction for Comparison of Algorithm Database; 3D, three-dimensional; SLIVER07, Segmentation of the Liver; ATLAS, Tumor and Liver Automatic Segmentation; CE-MRI, contrast-enhanced magnetic resonance imaging; HCC, hepatocellular carcinoma; CHAOS, Combined (CT-MR) Healthy Abdominal Organ Segmentation; DICOM, digital imaging and communications in medicine.

**Table 2:** Commonly Used Metrics for Liver Segmentation.

Metric	Description	Usage	Limitations
Dice similarity coefficient	Measure of similarity between predicted and ground-truth segmentations [0–1 (perfect similarity)]	Ideal for tracking model performance improvements	May be misleading due to imbalanced classes, heavier penalization of errors in smaller regions
Jaccard index	Ratio of intersection to union between predicted and actual segmentations	Commonly used to assess overlap and similarity	Sensitive to noise and minor boundary deviations, resulting in fluctuations
Accuracy	Proportion of correctly classified voxels out of the total number of voxels	Measures overall classification correctness	Insufficient alone for full evaluation of algorithm performance
Sensitivity	Ability to identify true-positive samples	Used to evaluate detection capability in relevant areas	Insufficient alone for full evaluation of algorithm performance
Specificity	Ability to correctly identify true-negative samples	Used to evaluate exclusion capability in irrelevant areas	Insufficient alone for full evaluation of algorithm performance
Volume overlap error	Quantification of error between predicted and ground-truth segmentations	Suitable for the evaluation of large-volume structures	May be inaccurate for small volumes and sensitive to incidental errors
Relative volume difference	Measure of the relative difference between predicted and actual volumes	Focuses on overall volume accuracy	Less effective for complex shapes and sensitive to noise
Average symmetric surface distance	Average distance between the boundaries of predicted and actual segmentations	Used to assess boundary accuracy and detail quality	May overemphasize minor boundary errors, neglecting overall segmentation accuracy
Maximum symmetric surface distance	Maximum distance between the boundaries of predicted and actual segmentations	Used to assess maximum boundary deviation	May overemphasize minor boundary errors, neglecting overall segmentation accuracy

**Table 3:** Overview of Liver Segmentation Techniques.

Method	Technique	Main Features	Advantages	Disadvantages
Manual segmentation		Liver contours delineated manually by radiologists or technicians	High accuracy, clinically accepted	Time consuming, operator dependent
Semi-automatic segmentation		Algorithm-based segmentation with manual input	Reduces manual workload, adapts to complex structures	Requires manual intervention for complex or atypical anatomy
	Threshold segmentation	Liver and non-liver tissues distinguished based on intensity threshold	Simple and fast, easy to implement	Sensitive to threshold setting, image quality, and noise
	Region-growing algorithm	Expansion from a seed point to include similar neighboring pixels	Adapts to local image variations, improves segmentation accuracy	Sensitive to initial point selection, limited for complex structures
	Edge-based segmentation	Edge detection algorithms used to identify liver boundaries	Effective for images with clear boundaries	Sensitive to noise and blurry edges, struggles with complex shapes
Fully automatic segmentation		Algorithm-driven segmentation with no manual intervention	Significantly reduces manual work, improves efficiency	Dependent on image quality and algorithm performance
	Edge-based segmentation	Edge detection algorithms used to identify liver boundaries	Effective for images with clear boundaries	Sensitive to noise and blurry edges, struggles with complex shapes
	Shape model segmentation	Statistical-shape or active-contour models used to guide segmentation	Enhances the recognition of complex structures, robust	High computational complexity, requires significant resources
	Fully convolutional network	Accepts arbitrarily sized images as input, makes pixel-level predictions via learned features	Handles complex image features	Less effective for small objects and detailed images
	U-Net	Symmetrical contracting and expanding paths used to capture context and fine details	Performs well on small datasets, captures context and details	Less effective for large images and complex background differentiation
	ResNet	Deep network models trained using residual connections	Improves model performance, supports deeper networks	Complex network structure, major hardware requirements, prone to overfitting on small datasets
	SegNet	Encoder–decoder structure, retains spatial information	Fewer parameters, suitable for fine segmentation tasks	Less accurate than U-Net and DenseNet for intricate medical images
	DenseNet	Efficient parameters, feature extraction improved with dense connections	Suitable for large 3D datasets	High computational cost, memory consumption
	V-Net	Specifically designed for 3D image data, segmentation accuracy enhanced through multiscale feature extraction	Adapts to 3D medical imaging data, captures spatial features	High computational resource requirement, long training times
	3D U-Net	Handles 3D image data, 3D contextual information utilized	Improves 3D image segmentation accuracy	High computational resource requirement, prone to overfitting on small datasets
	R2U-Net	U-Net, residual connections, and recurrent neural networks combined, enhancing feature extraction capabilities	Improves fine segmentation of complex organs, adapts to various liver sizes and shapes	Complex network structure, high computational resource demand
	Multimodal deep learning	Data from different imaging modalities integrated, enhancing diagnostic information	Improves diagnostic accuracy and reliability	Complex data alignment and processing, high computational resource requirement

## Data Availability

Not applicable.
